# sFlt-1/PIGF ratio positive associated with non-dipper type change in ambulatory blood pressure monitoring(ABPM) for preeclampsia development

**DOI:** 10.1038/s41440-023-01509-2

**Published:** 2023-11-28

**Authors:** Yunshan Chen, Xiaodan Di, Guochun Xiang, Yunfeng Liu, Xiuyu Pan, Wenfeng Deng, Xiongjie Zhu, Ming Lei, Guozheng Zhang, Huishu Liu

**Affiliations:** 1 Department of Obstetrics, Guangzhou Women and Children’s Medical Center, Guangzhou Medical University, Guangzhou, China; 2 School of Health Management, Southern Medical University, Guangzhou, 510515 Guangdong China; 3 Electrocardiographic Monitoring Unit, Guangzhou Women and Children’s Medical Center, Guangzhou Medical University, Guangzhou, 9 Jinsui Road, Guangzhou, 510623 China

**Keywords:** PE, Ambulatory blood pressure monitoring, sFlt-1/PIGF ratio, Non-dipper type, Nocturnal BP

## Abstract

In order to explore relationship of ambulatory blood pressure monitoring (ABPM) and soluble fms-like tyrosine kinase-1/placental growth factor (sFlt-1/PlGF) in suspected preeclampsia(PE), suspected PE participants in 28 + 0 to 33 + 6 weeks underwent ABPM and sFlt-1/PlGF from July 2020 to July 2022 were included(*N* = 476) in study. ABPM parameters were compared between sFlt-1/PlGF ≥38 and <38 groups. Correlation analysis was performed between ABPM and sFlt-1/PlGF, and logistic regression was used to explore prediction value for PE in 2 weeks. One hundred eighteen cases developed PE in 2 weeks with 114 from sFlt-1/PlGF ≥38 group. Daytime and nighttime BP were all increased,with increased non-dipper (58.4% vs. 30.3%), riser (22.1% vs. 13.1%) and and decreased Dipper (15.4% vs. 45.9%) type of ABPM in sFlt-1/PlGF ≥38 groups (*P* < 0.05).The riser group had the highest sFlt-1 and lowest PlGF. sFlt-1/PlGF and sFlt-1 were all positively correlated with systolic (SBP) & diastolic blood pressure(DBP)(*P* < 0.01), in which correlation coefficients of daytime and nighttime BP with sFlt-1 were *β* = 150.05 & 157.67 for SBP, *β* = 234 and 199.01 for DBP, respectively. However, PlGF was only negatively associated with nighttime SBP and DBP(*P* < 0.05), with no correlation with daytime BP (*P* > 0.05).Combining sFlt-1/PlGF and ABPM model, showed sFlt-1/PlGF (aOR = 2.01 (1.69–2.36)), Nighttime DBP (aOR = 1.14 (1.02–1.28)) contributed to preeclampsia prediction, and had improved predictive value compared to ABPM or sFlt-1/PlGF models alone(*P* < 0.05). sFlt-1/PlGF ratio was positively correlated with BP parameters, whereas PIGF was only negatively correlated with nocturnal BP and increased non-dipper type change in ABPM, which had a synergistic effect with sFlt-1/PlGF on PE prediction.

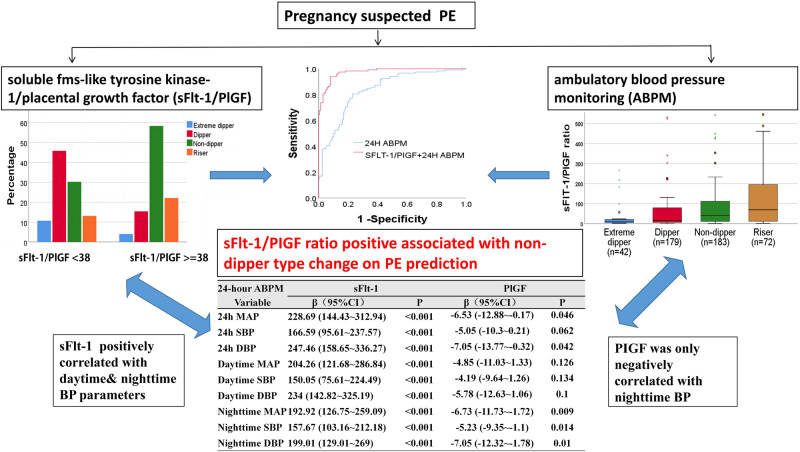

## Introduction

Preeclampsia (PE), a progressive multisystem disorder in hypertensive disorders of pregnancy, is characterized by the new onset of hypertension or significant end-organ dysfunction with or without proteinuria after 20 weeks of gestation or postpartum, which affects 2–8% of total pregnancies worldwide [1, 2].

PIGF(placental growth factor), secreted by syncytiotrophoblast, could promote trophoblasts proliferation, activation, while sFlt-1(soluble fms-like tyrosine kinase-1), the antagonist of PlGF, could block the biological activity of PIGF [3]. The unbalance of sFlt-1and PlGF was considered to reflect poor placentation of the spiral artery remodeling in PE pathophysiologic scheme [4]. Recent years, sFlt-1/PlGF ratio has been used to be a reliable index to predict PE in 1–4 weeks [5]. In the PROGOSIS research, the sFlt-1/PlGF ratio of 38 had a NPV of 99.3% (95% CI, 97.9–99.9) for PE, also with a PPV of 36.7% (95% CI, 28.4–45.7) [6], and the cutoff value was replicated in the Asian population across 25 centers [7].

While high blood pressure (BP) is the main earliest sign in PE development. Ambulatory Blood Pressure Monitoring (ABPM), a detailed BP monitoring method, provides a comprehensive record of all time BP levels and fluctuations within 24 h, which could identify “white-coat” hypertension and masked hypertension in pregnancy [8]. Types of BP variability indicated by ABPM (e.g., non-dipper type) have been associated with increased levels of endothelial inflammation cytokines, which might be reflective of small arterial spasms and inflammation [9].

As PE is considered to be a placental-origin inflammatory pathological disease, the sFlt-1/PlGF ratio and BP detailed parameters might both have changes in early development of PE, in which ABPM parameters and sFlt-1/PlGF values could indicate the severity of the inflammatory in peripheral and placental circulation levels separately.

Hence, a relationship and synergy between sFlt-1/PlGF ratio levels and ABPM parameters changes could be speculated in patients with suspected PE. In this research, 24-h ABPM parameters and levels of sFlt-1/PlGF in patients with suspected PE were collected, their changing trends and the collaborative relationships in PE prediction will be analyzed, in order to explore their interactive value for PE development.

Point of view

- Clinical relevance:
  sFlt-1/PlGF ratio is correlated with ABPM parameters changes, both of which have a synergistic effect on PE prediction.
- Future direction:
  Future research will further investigate the relationship between sFlt-1/PlGF ratio, ABPM, and adverse pregnancy outcomes and their intrinsic mechanisms in PE. This will help us further understand the significance of vascular abnormalities in maternal fetal interface and peripheral levels in hypertensive disorders.
- Consideration for the Asian population:
  Although there might be differences in the PE incidence and APPM diagnostic threshold for hypertension in pregnancy between Asian and other population, the synergistic relationship between sFlt-1/PlGF and ABPM parameters changes in PE development should be paid attention in Asian population.

## Materials and methods

### Study design and population group

This was a single-center observational study conducted from July 2020 to July 2022 at the Obstetric Department of Guangzhou Women and Children’s Medical Center, Guangdong, China. Pregnant women with suspected PE from 28 + 0 weeks to 33 + 6 weeks were enrolled in this study. Inclusion criteria included: (1) singleton pregnancy; (2) having new onset only one of suspicious symptoms but not reach PE diagnose criteria: hypertension, proteinuria, fetal growth restriction (FGR), decreased levels of platelets, increased liver enzymes, or edema. Exclusion criteria included: (1)multiple pregnancies; (2) confirmed PE diagnosis; (3) chronic hypertension; (4) have taking antihypertensive drugs; (5) concomitant chronic liver and kidney disease ;(6) lost follow-up in delivery.

Participants underwent both ABPM and sFlt-1/PlGF sampling at the time of initial inclusion. Maternal clinical characteristics including age, parity, height, weight, gestational age at the time of sampling were also collected. Participants received written information to donate their surplus aliquots of serum samples during prenatal care for scientific research before their written consent was obtained. This study was approved by the Institutional Ethics Committee of the Guangzhou Women and Children^’^s Medical Center (Reference number: 2017022008).

Participants were divided to two groups: sFlt-1/PlGF < 38 group and sFlt-1/PlGF ≥ 38 group, as sFlt-1/PlGF ratio 38 was considered a high NPV for PE. These two groups were followed up for 2 weeks from first inclusion to determine whether PE developed, and then followed until delivery. Delivery gestational weeks, peripartum maternal organ dysfunction levels, and fetal outcomes were also collected.

### Outcome definitions

PE refers to the new onset of hypertension accompanied by one or more of the following newonset conditions at ≥20 weeks’gestation (ISSHP diagnostic criteria): (1) proteinuria (2) other maternal end-organ dysfunction, including: (a) neurological complications (eclampsia, altered mental status, blindness, stroke, clonus, severe headaches, or persistent visual scotomata), (b) pulmonary edema, (c) hematological complications (platelet count < 150,000/μL, disseminated intravascular coagulation, hemolysis), (d) acute kidney injury (such as creatinine ≥ 90 μmol/L or 1 mg/dL), (e)liver involvement (alanine transaminase (ALT) or aspartate transaminase (AST) > 40 IU/l) with or without right upper quadrant or epigastric abdominal pain, (f)uteroplacental dysfunction (placental abruption, fetal growth restriction, abnormal umbilical artery Doppler waveform analysis, or intrauterine fetal death) [10].

### Serum sample collection and sFlt-1&PlGF measurement

A 5 ml peripheral maternal blood sample was obtained from patients with suspected PE at inclusion. The serum samples were separated by centrifugation at 2000 g for 10 min at room temperature and immediately stored at −80 °C until assayed at an independent laboratory. The levels of sFlt-1 and PlGF were measured using an electrochemiluminescence immunoassay analyzer (cobas e 411 system; Roche Diagnostics GmbH, Mannheim, Germany).

### Ambulatory blood pressure monitoring

Ambulatory BP was measured on the non-dominant arm during a 24-h period using an Oscar 2 ambulatory BP monitor (SunTech Medical). Measurements were recorded every 30 min from 6 am to 10 pm as daytime, and every 60 min from 10 pm to 6 am the following day as nighttime. During monitoring, the patients were instructed to (i) engage in normal activities, (ii) stop moving and talking during measurement, (iii) keep the arm still with the cuff at heart level at the time of cuff inflation, and (iv) record information on symptoms and events that may have influenced BP, meal times, in a diary. The measurements were downloaded to the computer and were considered technically satisfactory only if at least 85% of values during daytime and nighttime periods were satisfactory. The following values were obtained by ABPM: SBP(systolic blood pressure), DBP(diastolic blood pressure), HR(heart rate) in daytime, nighttime, 24 h period. The mean arterial pressure (MAP) was calculated as (SBP + (2 × DBP))/3 [11].

BP change pattern types groups of the ABPM: The nocturnal BP drop rates of 10%–20%, 0%–10%, <0%, and >20% were defined as dipper, non-dipper, riser, and extreme dipper types of ABPM, respectively. And the equation nocturnal blood pressure drop rate = (daytime average blood pressure value − nighttime average blood pressure value)/daytime average blood pressure value × 100% reflected the daytime and nighttime fluctuations of blood pressure.

### Statistical analysis

Maternal characteristics and perinatal period outcome data were compared between sFlt-1/PlGF <38 group and sFlt-1/PlGF ≥38 group. Data were expressed as mean ± standard deviation (SD) or median (25th–75th percentile as interquartile ranges) for continuous variables based on the distribution. Independent *t*-tests were used for normally distributed data, Mann–Whitney *U*-tests if otherwise. Counts with proportions were presented as frequencies and percentages, and tested using the chi-square (*χ*2) test.

A multivariable linear correlation analysis was conducted using Spearman correlation to assess the relationship between parameters of ABPM (mean blood pressure, systolic blood pressure, diastolic blood pressure, heart rate during daytime, nighttime, and 24 h) and sFlt-1, PlGF, as well as the sFlt-1/PlGF ratio. The results, including 95% confidence intervals (CI), were reported after adjusting by maternal characteristics(including maternal age, BMI, whether IVF, multiparity).

Binomial logistic regression analysis was used to generate a predictive model for PE by sFlt-1/PlGF, parameters of ABPM, and their combination, respectively. The area under curve (AUC) in receiver operating characteristic curve, were calculated to determine the performance of prediction model. For each of the models, sensitivity, specificity, positive predictive value (PPV), negative predictive value (NPV), were calculated. The power of the markers or parameters to increase prediction value was evaluated by discrimination (integrated discrimination index (IDI)) and reclassification analyses (free net reclassification index (NRI)).

All the analyses were performed with the statistical software packages R (http://www.R-project.org, The R Foundation) and *P* value of <0.05 was considered statistically significant.

## Results

### Description of the groups with suspected PE for different sFlt-1/PlGF levels

**Fig. 1:**
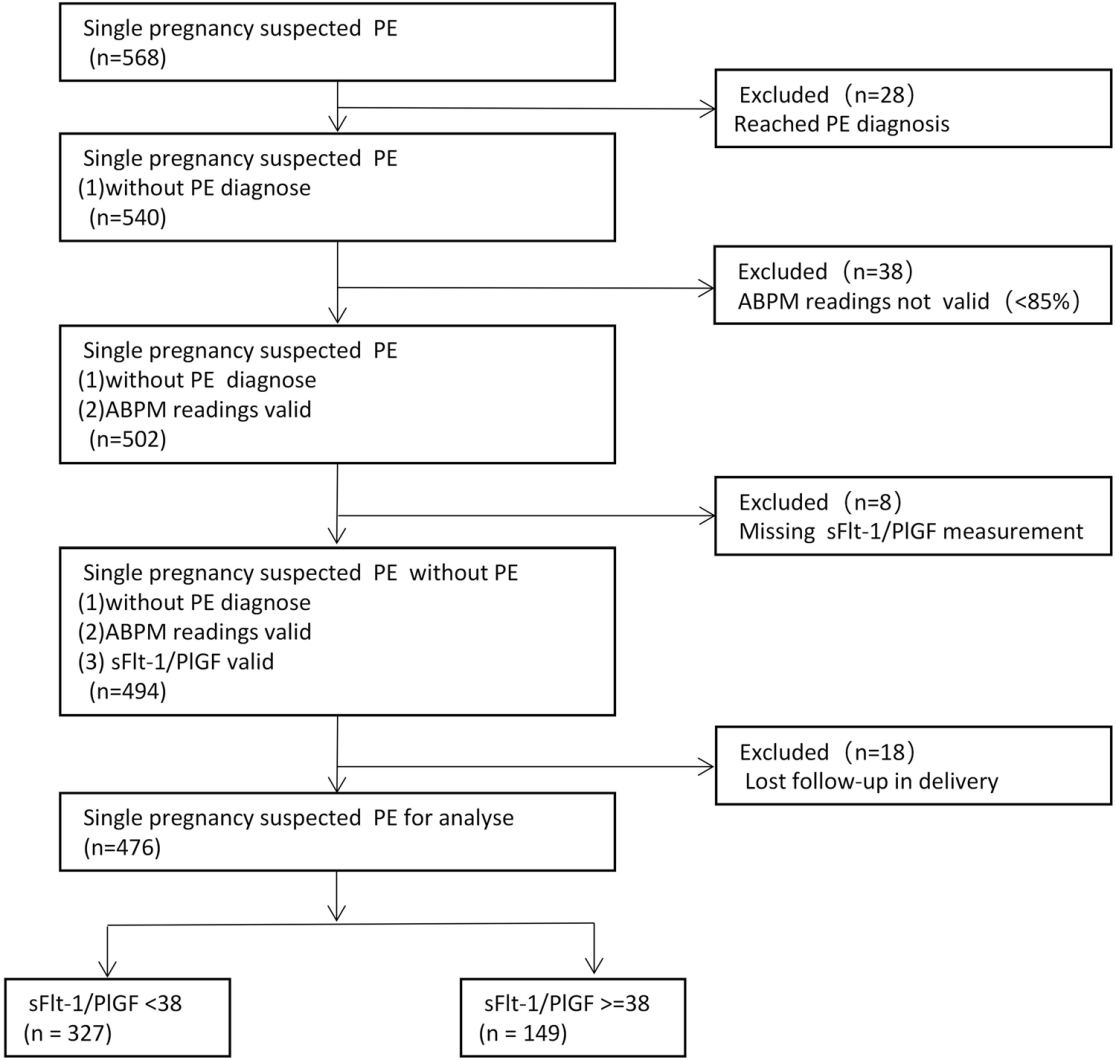
Study flowchart. ABPM ambulatory blood pressure monitoring, PE preeclampsia

A total of 568 pregnant women were enrolled. Women with <85% of valid ABPM readings or missing sFlt-1/PlGF data or reached PE diagnose or lost followup were excluded. Finally, 476 pregnant women with suspected PE were included in the analysis(Fig. 1), in which 149 cases sFlt-1/PlGF ≥38 and 327 cases sFlt-1/PlGF <38 as shown in Table 1. There were no statistical differences in terms of age, gravidity, and pregnancy complications (*P* > 0.05) between two groups, whereas a lower BMI and higher fetal growth restriction(FGR), IVF rate were observed in the sFlt-1/PlGF ≥38 group (P < 0.05).

**Table 1:** Clinical characteristics of the patients for different sFlt-1/PlGF groups

Variables	sFlt-1/PlGF < 38 (*n* = 327)	sFlt-1/PlGF ≥ 38 (*n* = 149)	*p*
Age (year)	31.9 ± 4.7	31.5 ± 5.2	0.464
BMI (kg/m²)	29.6 ± 4.0	28.3 ± 4.3	0.038
IVF, n(%)	13 (4%)	16 (10.7%)	0.004
Multiparity, n(%)	146(44.6%)	58(38.9%)	0.242
GDM, n (%)	104 (31.8%)	47 (31.5%)	0.955
Thyroid_disorders. n(%)	30 (9.2%)	18 (12.1%)	0.329
Rheumatoid_disorders, n(%)	8 (2.4%)	3 (2.0%)	0.911
FGR, n(%)	23 (7%)	40 (26.8%)	<0.001
ICP, n(%)	10 (3.1%)	6 (4%)	0.587
History of cesarean section, n(%)	49(15%)	20(13.4%)	0.654

*BMI* body mass index, *IVF* in vitro fertilization, *GDM* gestational diabetes, *FGR* fetal growth restriction, *ICP* intrahepatic cholestasis of pregnancy

114 cases from 76.5% of sFlt-1/PlGF ≥ 38 pregnancy developed PE in 2 weeks, while only 4(1.2%) of sFlt-1/PlGF < 38 cases developed PE(Table 2). And only 3 cases(sFlt-1/PlGF ≥ 38) developed PE 3–4 weeks after sampling. Levels of perinatal liver and kidney functions were significantly higher in the sFlt-1/PlGF ≥ 38 group, accompanied by an earlier gestational week of delivery, higher cesarean section rate, and lighter newborn weight (*P* < 0.05).

**Table 2:** Outcomes of the patients for different sFlt-1/PlGF groups

Variables	sFlt-1/PlGF < 38 (*n* = 327)	sFlt-1/PlGF ≥ 38 (*n* = 149)	*p*
PE in 2 weeks, n(%)	4 (1.2%)	114 (76.5%)	<0.001
Perinatal SBP(mmHg), Median (IQR)	141.0 (131.0, 147.0)	148.0 (140.0, 158.0)	<0.001
Perinatal DBP(mmHg), Median (IQR)	87.0 (81.0, 91.0)	92.0 (87.0, 100.0)	<0.001
Perinatal ALT (U/L), Median (IQR)	11.0 (9.0, 16.0)	14.0 (9.0, 19.0)	0.015
Perinatal AST (U/L), Median (IQR)	18.0 (15.0, 22.0)	22.0 (17.0, 26.0)	<0.001
Perinatal Cr(umol/L), Median (IQR)	46.0 (41.0, 53.0)	55.0 (48.0, 67.0)	<0.001
Perinatal UA(umol.L), Median (IQR)	351.0 (293.5, 410.0)	462.0 (386.0, 527.0)	<0.001
Gestational week in Termination (w)	37.4 ± 2.0	34.3 ± 3.4	<0.001
Cesarean section delivery. n(%)	189 (57.8%)	104 (69.8%)	0.013
Newborn weight(g), Median (IQR)	3070.0 (2820.0, 3305.0)	2360.0 (1720.0, 2925.0)	<0.001
SGA. n(%)	23 (7%)	40 (26.8%)	<0.001
Apgar1min < 7, n(%)	4 (1.2%)	25 (16.8%)	<0.001

*PE* preeclampsia, *SBP* systolic BP, *DBP* diastolic blood pressure, *ALT* alanine transaminase, *AST* aspartate transaminase, *Cr* creatinine,*UA* uric acid,*SGA* small for gestational age

### 24-h ABPM parameters in groups with for different sFlt-1/PlGF levels

**Fig. 2:**
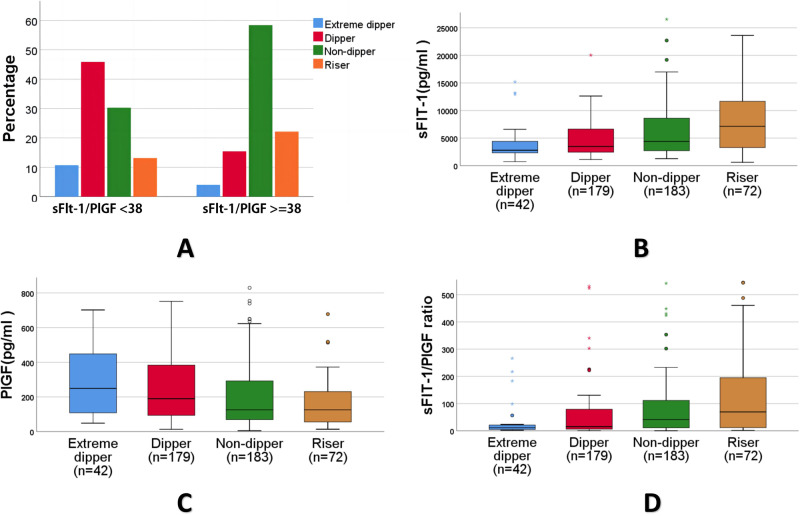
ABPM patterns in sFlt-1/PlGF group and sFlt-1/PlGF in different ABPM patterns. **A** Increased non-dipper and riser types were shown in the sFlt-1/PlGF ≥38 group with significantly lower dipper and extreme lower dipper types compared to sFlt-1/PlGF <38. **B**–**D** Among the different BP pattern types of the ABPM, the riser group had the highest sFlt-1 and the lowest PlGF with highest sFlt-1/PlGF ratio levels. In contrast, the dippers and extreme dippers groups had the lowest sFlt-1, and highest PlGF values with lowest sFlt-1/PlGF ratio levels

The 24-h, daytime and nighttime BP parameters were all significantly increased (*P* < 0.01), whereas the heart rate (HR) parameters were significantly decreased (*P* < 0.01) in the sFlt-1/PlGF ≥ 38 group. Increased non-dipper (58.4% vs. 30.3%) and riser (22.1% vs. 13.1%) types were shown in the sFlt-1/PlGF ≥ 38 group with significantly lower Dipper (15.4% vs. 45.9%) and Extreme lower Dipper types (4% vs. 10.7%)of ABPM fluctuation (*P* < 0.05) (Table 3, Fig. 2A).

**Table 3:** parameters of 24-h ABPM in different sFlt-1/PlGF levels

Variables	sFlt-1/PlGF < 38 (*n* = 327)	sFlt-1/PlGF ≥ 38 (*n* = 149)	*P*
24 h MAP(mmHg)	87.3 ± 8.6	94.2 ± 11.0	<0.001
24 h SBP(mmHg)	120.7 ± 10.5	127.9 ± 13.5	<0.001
24 h DBP(mmHg)	70.4 ± 7.9	77.3 ± 10.2	<0.001
Daytime MAP(mmHg)	88.6 ± 8.9	95.6 ± 11.0	<0.001
Daytime SBP(mmHg)	122.7 ± 10.2	129.2 ± 13.5	<0.001
Daytime DBP(mmHg)	72.3 ± 7.7	78.8 ± 10.2	<0.001
Nighttime MAP(mmHg)	81.6 ± 11.9	89.7 ± 12.7	<0.001
Nighttime SBP(mmHg)	114.6 ± 14.2	123.8 ± 15.6	<0.001
Nighttime DBP(mmHg)	64.9 ± 11.1	73.2 ± 12.2	<0.001
24 h HR(bpm), Median (IQR)	88.0 (81.0, 93.0)	80.0 (74.0, 88.0)	<0.001
Daytime HR(bpm), Median (IQR)	91.0 (84.0, 97.0)	83.0 (77.0, 90.0)	<0.001
Nighttime HR(bpm), Median (IQR)	81.0 (74.0, 86.0)	73.0 (68.0, 82.0)	<0.001
Extreme dipper type, n(%)	35 (10.7)	6 (4)	0.016
Dipper type, n(%)	150 (45.9)	23 (15.4)	<0.001
Non-dipper type, n(%)	99 (30.3)	87 (58.4)	<0.001
Riser type,n(%)	43 (13.1)	33 (22.1)	0.013

*MAP* mean BP, *SBP* systolic arterial BP, *DBP* diastolic BP, *HR* heart rate

Among the different BP pattern types of the ABPM, the riser group had the highest sFlt-1/PlGF ratio, highest sFlt-1 and the lowest PlGF value (Fig. 2B–D). In contrast, the dippers and extreme dippers groups had the lowest sFlt-1/PlGF ratio, lowest sFlt-1, and highest PlGF values.

### Association between 24-h ABPM and sFlt-1/PlGF ratio levels

The sFlt-1/PlGF ratio was all positively correlated with the daytime and nighttime MAP (*β* = 6.05 and 6.29), SBP (*β* = 3.96 and 4.68), and DBP (*β* = 7.62 and 6.64), respectively (*P* < 0.05), in ABPM (Table 4). In contrast, 24-h HR (*β* = −5.11), daytime HR (*β* = −5.28) and Nighttime HR(*β* = −3.62) were negatively correlated with the sFlt-1/PlGF ratio levels(*P* < 0.05).

**Table 4:** Multivariable linear correlation analyses for parameters of 24-h ABPM parameters with sFlt-1/PlGF levels

24-hour ABPM	sFlt-1/PlGF ratio		sFlt-1 level		PlGF level	
Variable	*β* (95%CI)	*P*	*β* (95%CI)	*P*	*β* (95%CI)	*P*
24 h MAP	7.04 (3.25–10.83)	<0.001	228.69 (144.43–312.94)	<0.001	−6.53 (−12.88–−0.17)	0.046
24 h SBP	4.6 (1.42–7.79)	0.005	166.59 (95.61–237.57)	<0.001	−5.05 (−10.3–0.21)	0.062
24 h DBP	8.06 (4.07–12.04)	<0.001	247.46 (158.65–336.27)	<0.001	−7.05 (−13.77–−0.32)	0.042
Daytime MAP	6.05 (2.34–9.75)	0.002	204.26 (121.68–286.84)	<0.001	−4.85 (−11.03–1.33)	0.126
Daytime SBP	3.96 (0.65–7.27)	0.02	150.05 (75.61–224.49)	<0.001	−4.19 (−9.64–1.26)	0.134
Daytime DBP	7.62 (3.55–11.68)	<0.001	234 (142.82–325.19)	<0.001	−5.78 (−12.63–1.06)	0.1
Nighttime MAP	6.29 (3.31–9.27)	<0.001	192.92 (126.75–259.09)	<0.001	−6.73 (−11.73–−1.72)	0.009
Nighttime SBP	4.68 (2.2–7.15)	<0.001	157.67 (103.16–212.18)	<0.001	−5.23 (−9.35–−1.1)	0.014
Nighttime DBP	6.64 (3.5–9.78)	<0.001	199.01 (129.01–269)	<0.001	−7.05 (−12.32–−1.78)	0.01
24 h HR	−5.11 (−8.98–−1.25)	0.01	−183.31 (−270.3–−96.33)	<0.001	13.77 (7.71–19.83)	<0.001
Daytime HR	−5.28 (−8.94–−1.62)	0.005	−182.41 (−264.53–−100.3)	<0.001	12.37 (6.58–18.16)	<0.001
Nighttime HR	−3.62 (−6.75–−0.49)	0.024	−98.89 (−188.68–−9.1)	0.02	10.42 (4.23–16.6)	0.001

Adjusted for maternal age, IVF, BMI

*MAP* mean BP, *SBP* systolic arterial BP, *DBP* Diastolic BP, *HR* heart rate

The sFlt−1 concentration also had strong positive associations with daytime and nighttime MAP (*β* = 204.26 and 192.92), SBP (*β* = 150.05 and 157.67), and DBP (*β* = 234 and 199.01), respectively (*P* < 0.01). However, the serum PlGF levels were only negatively correlated with nighttime MAP (*β* = −6.73), nighttime SBP (*β* = −5.23), and nighttime DBP (*β* = −7.05), (*P* < 0.05), but did not correlate with any daytime BP parameters (*P* > 0.05). Additionally, the PlGF concentration had strong negative associations with 24-h HR (*β* = 13.77), daytime HR (*β* = 12.37), and nighttime HR (*β* = 10.42) (*P* < 0.01).

### Synergistic value of 24-h ABPM and biomarkers (sFlt-1/PlGF) for preeclampsia prediction

**Fig. 3:**
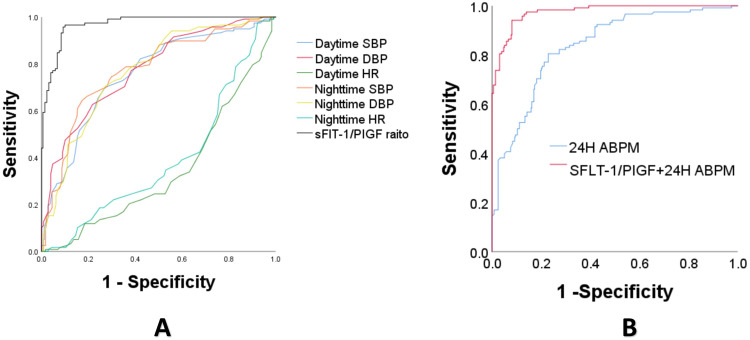
ROC curves of 24-h ABPM and sFlt-1/PlGF ratio for PE in 2 weeks. **A** AUC of ABPM parameters(SBP, DBP&HR) and sFlt-1/PlGF ratio for PE prediction in 2 weeks,separately. **B** AUC of combining the sFlt-1/PlGF ratio and ABPM parameters for PE in 2 weeks was 97.6%(96.3%–98.8%), significantly improved compared to 24-h ABPM model alone(SBP + DBP + HR (daytime + nighttime)+BP pattern types) with AUC 83.8%(95% CI 79.8%–87.9%) (*P* < 0.05)

When ABPM parameters (SBP + DBP + HR (daytime+nighttime)) all together to build model for PE in 2 weeks, the AUC could reach 83.8%(95% CI 79.8%–87.9%), although the individual ABPM parameter(SBP, DBP, HR) had a low AUC(Fig. 3A). For sFlt-1/PlGF ratio for predicting PE, the model AUC was 95.6%(94.7%–97.3%), with sFlt-1/PlGF 38 had NPV 96.3% and PPV 76.0%.

While combining sFlt-1/PlGF ratio and ABPM parameters (SBP + DBP + HR (daytime+nighttime)) together to build model, it showed improved predictive value as more AUC 97.6%(*p* < 0.05), RDI values 52.0% (40.7–63.3%)& 6.01% (1.02–10.7%), and IDI values 41.1% (35.2–46.9%) & 6.64% (3.85–9.43%), when compared to the ABPM or sFlt-1/PlGF models alone, respectively (*P* < 0.05)(Fig. 3B, Table 5). Moreover, the multiple logistic regression showed sFlt-1/PlGF ratio (aOR = 2.01 (1.69–2.36)), Nighttime DBP (aOR = 1.14 (1.02–1.28)) significantly contributed to PE prediction in this combined model(Table 6).

**Table 5:** Synergistic Value of ABPM and sFlt-1/PlGF for Preeclampsia Prediction

MODELS	AUC for PE (95%CI)	Sensitivity (95%CI)	Specificity (95%CI)	PPV (95%CI)	NPV (95%CI)	RDI(%) (95%CI)	IDI(%) (95%CI)
ABPM	83.8 (79.8–87.9)	39.83 (30.9–49.3)	94.97 (92.2–97.0)	72.7 (68.3–87.7)	83.0 (80.8–85.0)		
sFlt-1/PlGF	95.6 (94.7–97.3)	96.61 (91.5–99.1)	84.11 (85.4–92.1)	76.0 (68.4–79.8)	96.3 (96.8–99.5)		
sFlt-1/PlGF + ABPM	97.6 (96.3–98.8)	87.29 (79.9–92.7)	94.13 (91.2–96.3)	81.1 (76.3–88.2)	96.5 (94.2–97.9)	52.0 (40.7-63.3)^a^	41.1 (35.2-46.9)^a^
						6.01 (1.02-10.7)^b^	6,64 (3.85-9.43)^b^

*ABPM MODEL* SBP + DBP + HR (daytime + nighttime), *AUC* area under curve, *PPV* positive predictive value, *NPV* negative predictive value,*RDI* free net reclassification index *IDI* integrated discrimination index

^a^=sFlt-1/PlGF+ABPM MOEDL VS ABPM MODEL;

^b^=sFlt-1/PlGF+ABPM MOEDL VS sFlt-1/PlGF MODEL

**Table 6:** Multivariate logistic regression for PE in 2 weeks prediction from ABPM& sFlt-1/PlGF combined model

Variable	crude.OR_95CI	crude.P	adj.OR_95CI	adj.P
sFlt-1/PlGF	1.93 (1.68–2.22)	<0.001	2.01 (1.69–2.36)	<0.001
Daytime SBP	1.09 (1.06–1.11)	<0.001	1.01 (0.89–1.15)	0.869
Daytime DBP	1.14 (1.11–1.18)	<0.001	1.03 (0.93–1.13)	0.571
Nighttime SBP	1.06 (1.05–1.08)	<0.001	1.03 (0.98–1.11)	0.255
Nighttime DBP	1.08 (1.06–1.11)	<0.001	1.14 (1.02–1.28)	0.019
Daytime HR	0.95 (0.93–0.97)	<0.001	1.04 (0.95–1.14)	0.425
Nighttime HR	0.97 (0.95–0.99)	0.002	0.99 (0.9–1.08)	0.75

*PE* preeclampsia, *SBP* systolic arterial BP, *DBP* diastolic BP, *HR* heart rate

## Discussion

In our study, we found daytime and nighttime BP parameters, were both positively correlated with sFlt-1/PlGF ratio, whereas PIGF level was only negatively correlated with nocturnal but not daytime BP parameters, associated with increased non-dipper type change in ABPM.

Studies have shown that ABPM yields better sensitivity and specificity in the prediction of cardiovascular outcomes [12]. In pregnancy, ABPM riser types were more commonly observed in women with PE, which suggested that nighttime SBP and DBP failed to decrease during sleep (nocturnal hypertension) [13]. Normotensive women with a high-risk pregnancy presenting with nocturnal hypertension were found to have an increased risk of developing PE (OR 4.72, 95% Cl, 1.25–19.43) [14].

Interestingly, our findings showed that the serum PlGF level negatively correlated with nighttime without daytime BP parameters. Placental growth factor, a member of the vascular endothelial growth factor subfamily, secreted by the placenta, was found abnormally reduced in the maternal blood circulation of PE patients and much earlier than the onset of PE [15]. Studies have shown that PlGF, which promotes proliferation and reduces apoptosis of trophoblasts, reflects the degree of placental angiogenesis [16]. The negative correlation between PIGF and nighttime BP observed in our study also suggested that nighttime BP was an important indicator of basal placental circulation, which is the foundation of PE pathogenesis. Therefore, concerning placental circulation, the importance of nocturnal hypertension in the prediction and evaluation of PE should be further addressed.

Soluble fms-like tyrosine kinase-1, which binds to and reduces the level of free pro-angiogenic factor PlGF, contributes to the inflammatory changes in PE [17]. Different thresholds of sFlt-1/PIGF ratios, an index of placental inflammation, have been explored in previous studies to predict the occurrence and severity of PE [18]. Verlohren et al. found that pregnant women with a high sFlt-1/PlGF ratio had more adverse maternal and fetal outcomes such as placental abruption, elevated liver enzymes, or FGR [19]. The positive correlations between sFlt-1 and both daytime and nighttime SBP, DBP, and HR in the current study indicated that regardless of the susceptibility to external factors, daytime BP changes were also indicative of the placental inflammatory response.

The correlation between ABPM parameters, especially nocturnal BP, and the sFlt-1/PIGF ratio indicated a synergy of the two in the prediction of PE development. Niu et al. used ABPM to explore the thresholds of BP parameters based on perinatal outcomes and achieved good sensitivity and specificity [20]. Also, sFlt-1/PIGF plus the traditional diagnostic PE criteria could significantly improve the sensitivity to predict adverse perinatal outcomes [21, 22]. In our study, when combining the sFlt-1/PIGF and ABPM parameters together, high AUC was obtained in PE prediction model with sFlt-1/PlGF ratio (OR = 2.11 (1.69–2.36)) & Nighttime DBP (OR = 1.14 (1.02–1.28)), which suggested the importance of multi-aspect comprehensive evaluation for PE prediction, including peripheral and placental circulation levels.

While our study still had a few limitations. Due to the inclusion criteria of high-risk individuals with suspected PE, the incidence of PE in the final included population was 24.7%, which was different from the 2–8% incidence of PE in the general population. Also, the population sample was only from single-center design. These may lead to a research population specific bias in this study, and the conclusion need further validation for women with low-risk pregnancies. And as the time interval in sampling to delivery differences in cases, the relationship of BP and sFlt-1/PIGF levels might have some changes in prenatal period. Future multi-center studies and better research design are needed to confirm our findings.

### Perspective of Asia

The relationship between sFlt-1/PlGF and ABPM parameters changes illustrated the synergistic value of ABPM in PE prediction for Asian population, while the sFlt-1/PlGF ratio has shown its value in the PROGNOSIS Asia study [7]. However, there might be differences in the PE incidence and APPM diagnostic threshold for hypertension between Asian and other population [1, 8]. Thus, further prospective study is required to confirm the their association significance for Asian individuals.

## Conclusion

The sFlt-1/PlGF ratio was positively correlated with ABPM BP parameters, whereas PIGF concentration was only negatively correlated with nocturnal hypertension, associated with increased non-dipper type change shown in ABPM. Combining sFlt-1/ PIGF ratio with ABPM parameters, especially nocturnal BP, synergistically contribute to PE prediction, which could be significant for the future multi-dimensional PE early prognose and management strategy.
